# State-of-the-Art Applications of Field-Effect Transistor Biosensors in Exosome Detection: A Comprehensive Review

**DOI:** 10.3390/bios16050294

**Published:** 2026-05-18

**Authors:** Xinyi Sheng, Guo-Jun Zhang, Jie Zhou

**Affiliations:** 1 School of Laboratory Medicine, Hubei University of Chinese Medicine, Wuhan 430065, China; 2432800020@stmail.hbucm.edu.cn; 2 Hubei Shizhen Laboratory, Wuhan 430065, China

**Keywords:** field-effect transistor, biosensor, exosome detection, isolation, disease diagnosis

## Abstract

Exosomes are a kind of nanoscale extracellular vesicle secreted by almost all cell types and considered promising biomarkers for disease diagnosis since they could carry abundant proteins, nucleic acids, and lipids that reflect parental cell states. However, conventional exosome detection methods suffer from several limitations including insufficient specificity, low throughput, high costs, and inadequate sensitivity for clinical applications. By contrast, field-effect transistor (FET) biosensors are a promising alternative by enabling label-free, real-time, and ultrasensitive detection of exosomes through direct transduction of biorecognition events into electrical signals. This review first introduces the fundamental principles and device structure of FET biosensors, as well as exosome isolation strategies. The recent advances in exosome analysis using FET-based biosensors are then presented, which are categorized into two primary strategies: (1) direct detection of intact exosomes based on surface markers, including tetraspanin proteins (CD9, CD63, CD81, etc.) and disease-specific biomarkers, and (2) detection of exosomal contents including microRNA and protein biomarkers following exosome lysis. Finally, we discuss current challenges of FET-based exosome detection and provide perspectives on future developments.

## 1. Introduction

Exosomes are a class of extracellular vesicles (EVs) secreted by almost all cell types with diameters ranging from 30 to 150 nm. These nanoscale vesicles carry various biomolecules such as proteins, lipids, DNA, mRNA, and microRNAs, which are derived from their parent cells [1]. Exosomes play essential roles in intercellular communication, angiogenesis, tumor progression, and metastasis. Therefore, they act as key mediators in both physiological and pathological processes [2,3]. Exosomes have attracted considerable attention as disease biomarkers due to several outstanding advantages [4,5]. First, the lipid bilayer membrane of exosomes effectively protects the nucleic acids and proteins inside from enzymatic degradation in complex biological environments [6]. Second, exosomes are widely distributed in multiple body fluids at relatively high concentrations (10^6^–10^11^ particles/mL) [7]. And the exosome isolation method has been increasingly well established [8]. Third, unlike single molecular markers, exosomes carry diverse molecules derived from their parental cells, which provide richer diagnostic information [9].

Although exosomes have significant potential as biomarkers, the detection and characterization of exosomes is still challenging [10]. Current methods for exosome detection rely primarily on the physical properties, surface markers, or cargo molecules of exosomes [11]. Nanoparticle tracking analysis (NTA) is used to determine the size distribution and concentration data of exosomes. However, it lacks the ability to distinguish exosomes from other co-isolated particles like lipoproteins [12]. Transmission electron microscopy (TEM) provides high-resolution nanoscale imaging of exosomes. But it has the drawbacks of labor-intensive sample preparation and low throughput [7]. More importantly, neither NTA nor TEM can achieve accurate qualitative and quantitative detection of exosome subpopulations. Conventional flow cytometry can perform multiparametric phenotyping of exosomes. However, the detection threshold of 200–300 nm limits the analysis of smaller vesicle subpopulations [13]. Quantitative approaches also have inherent limitations. Immunological quantitative methods such as ELISA and Western blotting have inherent shortcomings, including insufficient sensitivity(~10^7^ particles/μL) [14], being only semi-quantitative [15], and poor repeatability. Meanwhile, omics technologies like qRT-PCR, NGS, and LC-MS/MS, although capable of achieving fine mapping analysis of exosome contents, are highly dependent on complex sample pretreatment, expensive professional instruments, and personnel, with long detection cycles and high costs [16,17], thus failing to meet the core requirements of rapid and point-of-care clinical testing (POCT). All the above shortcomings have restricted the reliable and efficient application of exosomes in clinical practice and scientific research. It is therefore necessary to develop new detection technologies that can overcome these drawbacks and fulfill the potential of exosomes as biomarkers.

**Figure 1:**
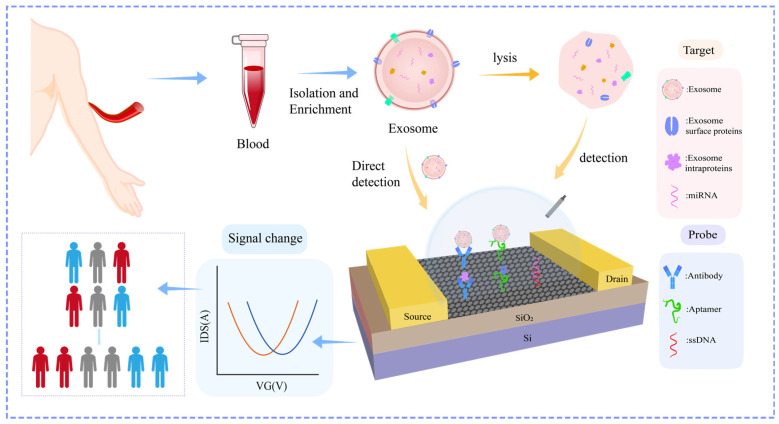
A simplified scheme of an FET biosensor for exosome detection.

Field-effect transistor (FET) biosensors are recognized as a promising platform for exosome analysis [18], with multiple core advantages that address the limitations of conventional detection methods. These devices directly transduce biorecognition events into measurable electrical signals through real-time monitoring of biomolecular interaction-induced electric field changes, realizing label-free and rapid detection of exosomes [19]. Meanwhile, FET biosensors exhibit ultrahigh sensitivity, enabling stable detection of trace exosomal targets in complex clinical samples. They also feature low cost and excellent compatibility with miniaturization and integration, which can get rid of bulky instruments and show great potential for clinical POCT [20]. Owing to these outstanding advantages, FET sensors based on various semiconducting materials and device structures have been successfully developed for exosome detection, which are mainly divided into two categories (Figure 1): one is direct capture and quantification of intact exosomes by functionalizing the sensing surface with recognition elements targeting membrane proteins [21], and the other is detection of nucleic acid and protein biomarkers in exosome lysates.

In recent years, researchers have employed various strategies to enhance the performance of FET biosensors for exosomes. These include: (i) the development of novel nanoscale channel materials and modification strategies to optimize carrier transport properties and biorecognition efficiency [22,23,24,25,26,27]; (ii) the integration of multifunctional microfluidic systems to realize one-stop processing of exosome isolation, enrichment and in situ detection [28,29]; (iii) the introduction of machine learning to achieve high-accuracy intelligent disease diagnosis and result interpretation [30]; (iv) the development of miniaturized, low-power integrated detection platforms to support clinical POCT [31,32]; and (v) the development of wafer-scale fabrication of nanoscale channel material to ensure the high uniformity of sensors [33]. Together, these approaches contribute to enhancing the sensitivity and specificity of biosensors while expanding their applications in exosome detection.

Although there have been numerous reviews focusing on the biomedical and biomarker detection applications of FET biosensors, reviews focusing on the application of FET sensors in exosome detection are still lacking. In this paper, we review the advancement of FET biosensors in exosome analysis in recent decades. Firstly, we introduce the basic principles and structure of FET biosensors. Then, current methods for exosome isolation and enrichment are summarized. In the third section, the recent advances in FET-based detection of both intact exosomes and exosome-derived biomarkers are presented in detail. Finally, we conclude with the challenges and future perspectives of FET biosensors in exosome analysis.

## 2. Principles of Field-Effect Transistor Biosensors

### 2.1. Basic Operating Mechanism and Architectures of FET Devices

#### 2.1.1. Basic Operating Mechanism of FET Devices

FET biosensors are a class of novel biochemical sensing devices based on the semiconductor field-effect principle, and their core operating mechanism builds on the charge modulation characteristics of metal-oxide-semiconductor (MOS) FETs. A MOSFET consists of four fundamental components: source, drain, gate, and a semiconductor channel between the source and drain. The gate is separated from the semiconductor by a thin insulating oxide layer, forming a MOS capacitor structure [34]. In a typical n-channel enhancement-mode MOSFET, the source and drain are two heavily doped n-type regions formed by diffusion into a p-type silicon substrate, and the p-type region between the source and drain acts as the channel [35]. When no voltage is applied to the gate, the source and drain are isolated by two back-to-back p-n junctions, which prevent the formation of a conductive pathway [36]. Upon application of a positive gate voltage, positive charges would accumulate on the gate electrode. Then, through the capacitive coupling across the oxide layer, negative charges are induced at the semiconductor surface adjacent to the oxide [37]. As the gate voltage increases, holes in the p-type semiconductor surface are repelled into the bulk substrate, while electrons in the substrate accumulate at the surface. When the electron concentration at the surface exceeds the hole concentration, the region forms an n-type conductive channel between the source and drain [38]. And a substantial drain current is generated under the applied source–drain voltage [39]. Consequently, the gate voltage directly determines the density of induced charges in the channel and precisely controls the channel conductance and source–drain current, which is the origin of voltage-controlled current amplification and switching functions [40].

#### 2.1.2. Architectures of FET Devices

Based on the position, configuration, and structure of the gate electrode, FET sensors can be classified into top-gate, back-gate, liquid-gate, and extended-gate types.

**Figure 2:**
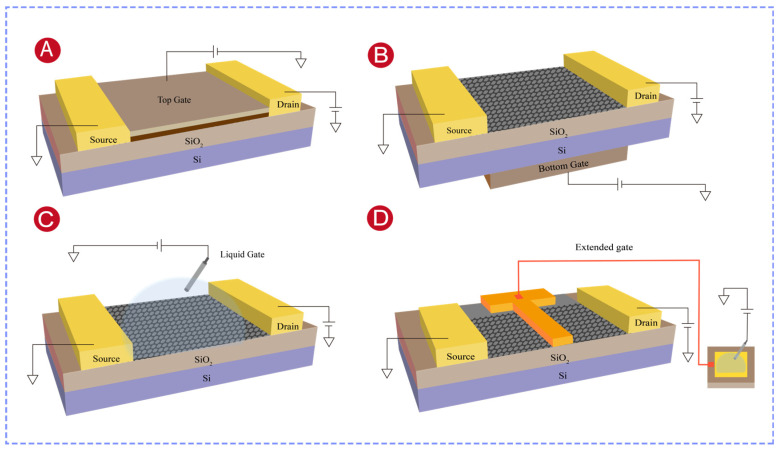
Different FET sensors based on the configuration of the gate electrode. (**A**) Top-gated FETs. (**B**) Back-gated FETs. (**C**) Liquid-gated FETs. (**D**) Extended-gate FETs.

In top-gate FETs, the gate electrode is positioned directly above the channel and separated from it by an insulating oxide (Figure 2A). When a top-gate voltage is applied, an electric field perpendicular to the channel is generated and modulates the carrier concentration in the channel [41]. This represents the conventional MOSFET configuration. To enhance the efficiency of gate control, high-κ dielectrics (such as HfO_2_ and Al_2_O_3_) have been introduced. Their higher dielectric constants produce stronger electric fields at the same gate voltage, which could lower the threshold voltage, improve the gate control efficiency, and reduce the leakage current [42]. However, top-gate fabrication is relatively complex and costly. Additionally, the interface between the gate electrode and the dielectric layer is susceptible to charge trapping induced by radiation or bias stress, which would reduce the long-term stability of the device [43,44].

In back-gate FETs, the gate electrode is positioned beneath the channel and forms a capacitive coupling with the channel material through a buried oxide layer. The carrier concentration is modulated by applying a back-gate voltage (Figure 2B) [45]. This configuration offers a simpler structure and easier fabrication [46]. However, the gate control efficiency of back-gate devices is generally lower than that of top-gate devices due to the greater gate-to-channel distance [47].

Liquid-gate FETs employ a reference electrode to apply the gate voltage and use an electrolyte solution as the gate dielectric (Figure 2C). Carrier modulation is achieved through the electrical double layer formed at the interface between the electrolyte and the channel material [48,49]. Because the electrolyte conforms closely to the channel surface, the interface defects of solid-state gates are minimized. This would reduce the defect-induced carrier scattering and substantially enhance the carrier mobility [50]. The low-dimensional geometry and unique physicochemical properties of nanomaterials are well-suited to the operational requirements of liquid-gate FETs, which improve the sensitivity, response speed, and specificity of sensors, and expand their applications in various fields.

Extended-gate FETs and floating-gate FETs share a common design philosophy: in both architectures, the sensing region is spatially separated from the transistor channel, which protects the signal transduction unit from solution corrosion and ionic interference. The two architectures, however, differ in how the interfacial potential generated by target binding is transmitted to the transistor. In extended-gate FETs, the sensing electrode is electrically connected to the gate of the transistor through a metal wire (Figure 2D), so that charge variations at the recognition interface [51] directly modulate the gate voltage and thereby alter the source–drain current [52]. In floating-gate FETs, by contrast, the sensing electrode (control gate) is capacitively coupled to an electrically isolated floating gate embedded within the device, with no ohmic connection between them. Target-induced potential changes are transferred to the floating gate across a dielectric layer, which in turn modulates the channel current. The primary advantage of extended-gate FETs is that only the extended gate requires replacement for repeated use and the performance of the signal transduction unit would not be affected by solution corrosion or ionic interference. However, after several uses, the detection accuracy would possibly be reduced by the signal drift [53]. Furthermore, extended-gate FET fabrication requires complex manufacturing processes and relatively high costs.

The properties of channel materials are critical to the performance of FET sensors. Physical characteristics such as carrier mobility, electrical conductivity, and bandgap directly influence the response speed and detection sensitivity. And chemical properties such as the type and density of surface functional groups, hydrophilicity/hydrophobicity, and chemical stability affect the immobilization efficiency, activity, and stability of the probe. In terms of channel material, three main categories have been established in the field of FET biosensing: bulk inorganic semiconductors, nanomaterials, and organic/polymer semiconductors. Amorphous oxide semiconductors such as indium gallium zinc oxide (IGZO) have also been explored as channel materials. IGZO films are transparent and can be deposited at low temperatures [54]. Their surface can be functionalized through self-assembled monolayers (SAMs) to enable selective binding of exosome biomarkers [31]. IGZO-based FETs show good electrical stability in aqueous environments, making them suitable for biosensing applications.

For bulk inorganic semiconductors, silicon is the most widely used channel material in FET biosensors. It has well-established electrical properties and is compatible with standard microfabrication processes [55]. The surface of silicon can be easily modified with silane chemistry, which allows the attachment of antibodies, aptamers, or other recognition molecules. However, bulk silicon has a relatively low surface-to-volume ratio, which limits its sensitivity compared to nanoscale materials [56]. In recent years, advances in nanotechnology have led to the widespread use of nanomaterials such as silicon nanowires [57], carbon nanotubes [58], and graphene [59] in channel construction. These nanomaterials have ultrahigh surface-to-volume ratios, excellent carrier transport properties, easy surface functionalization, and unique electronic structures. As a result, the nano-FET biosensors could achieve ultrasensitive detection, rapid response, and multiplexed analysis of target analytes [60]. In exosome detection, nano-FET biosensors show good application potential and may become a core technology for building next-generation high-performance sensing platforms. Nanomaterials can be simply divided into one-dimensional (1D) and two-dimensional (2D) materials.

FETs based on 1D nanomaterials have been extensively applied and investigated owing to their advantages of large specific surface area and ultrahigh sensitivity [61]. The most representative ones are silicon nanowire (SiNW) FETs and carbon nanotube (CNT) FETs, where the channel diameter of SiNW FETs is typically 10–100 nm. On the surface of SiNW channels, the specific recognition and binding between charged target molecules and the functionalized sensing interface triggers the depletion or accumulation of carriers in the channel, thereby regulating the electrical transport behavior of the nanowire FET and generating a local signal response [62]. The unique structure of nanowires resembles a fully wrapped gate configuration, in which the biological medium surrounding the nanowires can act as a gate to modulate the channel, exerting an extreme gate control coupling effect. Furthermore, the characteristic size of nanowires matches that of most biological units, endowing them with excellent dimensional compatibility. The surface of SiNWs is rich in hydroxyl groups, which can be readily modified with silane agents to attach recognition molecules such as antibodies or aptamers. In addition to silicon, metal oxide nanowires such as zinc oxide (ZnO) and indium oxide (In_2_O_3_) are also used as channel materials for FETs.

CNT FETs combine the merits of nanowires (large specific surface area) with the advantages of atomic-scale thickness and high carrier mobility, conferring exceptional electrical driving capability and electrostatic control capability [63]. Moreover, CNTs can be fabricated into aligned arrays and network structures, showing great potential for miniaturization and large-scale production. Common single-walled CNTs (SWCNTs) can exhibit metallic or semiconducting characteristics due to differences in their chirality. However, metallic CNTs are unsuitable for FET applications because their conductive properties are difficult to modulate. Therefore, the construction of high-performance CNT FETs necessitates the use of high-purity semiconducting CNTs. The surface of CNTs can be modified through non-covalent interactions (such as π–π stacking) or covalent functionalization to attach recognition elements.

Among 2D nanomaterial-based FETs, the graphene FET is the most representative. It is composed of a monolayer of carbon atoms arranged in a 2D lattice, with all carbon atoms fully exposed to the ambient environment. Endowed with the inherent advantages of an atomically thin layered structure and ultrahigh specific surface area, it enables highly sensitive detection of biomolecules [64]. Graphene is intrinsically bandgap-free, which imparts its transfer curve with a characteristic Dirac point, where the conductance reaches the minimum [65]. Reduced graphene oxide (rGO) can also serve as the channel material for FETs, exhibiting properties comparable to those of pristine graphene. However, in contrast to pristine graphene, rGO possesses intrinsic surface structural defects and abundant chemical functional groups, rendering it more favorable for the functional modification of biomolecules [66]. Graphene can be functionalized via non-covalent modification (e.g., π–π stacking with aromatic molecules) or covalent modification following oxidation to graphene oxide (GO). Beyond these two materials, transition metal dichalcogenides (TMDs, such as MoS_2_, WS_2_, WSe_2_, and MoTe_2_), hexagonal boron nitride (h-BN), black phosphorus (BP), and transition metal oxides have been extensively investigated and employed as channel materials for FETs.

Organic semiconductors such as PEDOT:PSS and P3HT are commonly used in OECTs [67]. These materials can operate in aqueous solution, which makes them naturally compatible with biological samples. PEDOT:PSS, in particular, is a mixed ionic–electronic conductor [68]. It can transduce ionic signals from the biological environment into electronic signals, which amplifies the sensing response. The surface of organic semiconductors can be modified with charged polymers or biomolecules to achieve selective binding. Compared to inorganic materials, organic semiconductors are more flexible and can be deposited on soft substrates, which is attractive for wearable or implantable biosensing applications [69].

### 2.2. Working Mechanisms of FET Biosensors for Exosome Detection

FET biosensors are widely used for quantitative biomolecule detection because they offer high sensitivity, fast response, and label-free measurement. In general, they work by converting biomolecular binding events at a solid–liquid interface into measurable electrical signals. In this section, we describe the general working mechanism of FET biosensors and discuss the key factors relevant to exosome detection.

Device configuration and the signal transduction chain. A typical FET biosensor consists of a semiconductor channel, source–drain electrodes, a gate electrode and recognition elements modified on the sensing interface (e.g., antibodies, nucleic acid aptamers, or peptide ligands). In most biosensing configurations, a liquid-gate design is used, where a reference electrode serves as the gate and the measurement buffer acts as the electrolyte. At both interfaces, the channel surface/electrolyte interface and the gate electrode/electrolyte interface, an electrical double layer (EDL) forms, comprising a Stern layer and a diffuse layer [70]. These EDLs enable electrostatic coupling within the device [71]. The EDL capacitances at the channel side (*C*_dl,ch_) and gate side (*C*_dl,g_) are connected in series to define the total system capacitance *C*_total_:

1/*C*_total_ = 1/*C*_dl,ch_ + 1/*C*_dl,g_ (1)

When target biomolecules in the sample bind specifically to the receptors on the sensing surface, the resulting change in surface charge density (Δσ) modifies the local surface potential (ψ_0_) through the channel-side EDL capacitance:

Δ*ψ*_0_ ≈ Δσ/*C*_dl,ch_ (2)

This potential shift acts as an effective gate voltage modulation (Δ*V*_g_^eff^), which translates into a measurable drain current change governed by the device transconductance g_m_:

Δ*I*_DS_ ≈ *g*_m_ × Δ*V*_g_^eff^ (3)

In graphene FETs this appears as a shift of the Dirac point, while in other FETs it manifests as a threshold voltage shift. Equations (2) and (3) show that the signal magnitude is determined not only by the amount of bound target, but also by how efficiently the bound charges couple to the channel through the EDL.

Electrostatic gating. Among all transduction pathways, electrostatic gating is the dominant mechanism in most exosome detection studies. Exosomes typically carry a negative surface charge, with zeta potentials between −10 and −30 mV. When they bind to the sensing surface, they increase the net negative surface charge density and shift *ψ*_0_ toward more negative values. For n-type FETs, a more negative *ψ*_0_ reduces electron accumulation in the channel and lowers conductance. For p-type FETs, the same shift leads to a rise in conductance, while positively charged targets produce inverted responses [72].

Debye screening and its implications for the two detection strategies. The most important interfacial parameter governing FET sensitivity in exosome sensing is the Debye length:

*λ*_D_ = √[*ε*ε_0_*k*_B_*T*/(2*N*_A_*e*^2^*I*)] (4)

which describes the distance over which charge perturbations decay in an electrolyte. In undiluted physiological buffers (1× PBS, *I* ≈ 162 mM), *λ*_D_ is only ~0.7–1 nm; diluting to 0.01× and 0.001× PBS extends *λ*_D_ to approximately 7 nm and 24 nm, respectively [73]. This single parameter governs the sharply different signal behaviors of the two main exosome detection strategies.

In intact-exosome detection, the analyte is a 30–150 nm vesicle whose diameter far exceeds *λ*_D_ under physiological conditions. When a vesicle is captured by a surface-immobilized probe, only the charges on the part of the vesicle closest to the channel contribute to Δ*V*_g_^eff^, while charges on the far side are electrostatically screened [74]. The signal therefore originates mainly from membrane-associated charges near the binding interface, rather than from the vesicle as a whole.

In lysate-based detection, by contrast, the disrupted vesicles release miRNA or protein targets that bind directly to surface-anchored probes. These small targets sit within a few nanometers of the channel, well within *λ*_D_, so Debye screening is far less limiting and direct electrostatic gating dominates [75].

EDL restructuring and capacitance modulation. Beyond simple charge screening, target binding restructures the interfacial EDL through ion redistribution, dipole reorientation, and steric displacement of water and counterions [76]. These effects modify both *C*_dl,ch_ and *C*_dl,g_, and hence *C*_total_. This pathway is particularly relevant to intact-exosome detection. Even when the net charge signal is reduced by Debye screening, the captured vesicle physically disrupts the EDL over an area comparable to its size, producing a capacitance change that contributes to the measured response.

Concurrent mechanisms in the two detection strategies. In practice, these mechanisms rarely act in isolation. In intact-exosome detection, a single binding event may simultaneously generate a screened electrostatic signal and an EDL restructuring contribution, which partly explains why intact-exosome assays tend to show greater device-to-device variability. In lysate-based detection, direct electrostatic gating dominates because the small targets lie within *λ*_D_, making signals more consistent. Attributing the response of a FET exosome sensor to a single mechanism is therefore generally an oversimplification, and signal interpretation should take into account the specific detection strategy, device architecture, channel material, and measurement protocol.

## 3. Exosome Isolation and Enrichment Methods

While exosomes have strong potential as biomarkers, a primary challenge in detecting them is efficiently isolating and enriching these vesicles from complex biological matrices [77]. Body fluids such as blood, urine, and saliva contain many other components besides exosomes, and they may share similar physical features with exosomes, which increases the difficulty of separating them [78]. As a result, current isolation approaches often produce low-purity samples, which can reduce the reliability of results [79]. In addition, exosomes are highly heterogeneous, with different subpopulations varying in size, density and surface markers, which increases the isolation complexity [80]. Therefore, isolation strategies are selected for the intended application, with careful trade-offs between yield, purity, vesicle integrity and practicality. This section overviews the methods for exosome isolation and enrichment, mainly including the methods based on the physical properties of exosomes, immunoaffinity-based methods and microfluidics-based methods.

### 3.1. Isolation and Enrichment Methods Based on the Physical Properties of Exosomes

Physical property-based methods separate and enrich exosomes by exploiting the differences in density, sedimentation coefficient, size, and hydrodynamic behavior between exosomes and other components in samples [81]. These methods do not rely on chemical or biological recognition. The main techniques include ultracentrifugation [81], ultrafiltration [82], size exclusion chromatography [83] and polymer precipitation.

#### 3.1.1. Ultracentrifugation

**Figure 3:**
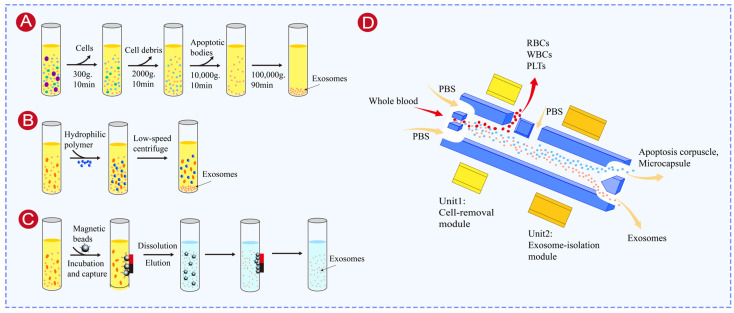
A simplified scheme of ultracentrifugation (**A**), polymer precipitation (**B**), immunoaffinity (**C**), and microfluidics (**D**) based isolation and enrichment methods [88]. Copyright (2017) National Academy of Sciences.

Ultracentrifugation separates exosomes from other components based on differences in sedimentation coefficient, which is related to particle size and density. It is widely used because it is easy to perform and inexpensive [84]. This method first removes unwanted components through low- and medium-speed centrifugation, followed by high-speed centrifugation at 110,000× *g* to collect purified exosome pellets [85] (Figure 3A). However, this approach has some drawbacks: it requires large sample volumes to get enough exosomes, and a single run can take over 6 h. The high centrifugal forces can also damage the exosome membranes or cause aggregation, which can affect later analysis [86]. Density gradient centrifugation is an improved version of ultracentrifugation. By using density gradients like sucrose, exosomes (with a density of 1.13–1.19 g/mL) are concentrated in specific density zones during centrifugation, separating them from similar-density contaminants like lipoproteins. This method gives purer exosomes and can even isolate specific exosome subgroups [87]. However, it requires additional preparation of gradient media, increases centrifugation time (usually 12–24 h), and needs careful control of temperature and rotor speed, which depends on more precise equipment and increases the batch variability.

#### 3.1.2. Ultrafiltration

Ultrafiltration is a size-based method for exosome separation and enrichment. It uses membranes with a defined molecular-weight cut-off (MWCO) value, and centrifugal force pushes samples through the membranes. Exosomes could be separated and enriched by two-step ultrafiltration. First, positive ultrafiltration is used to make exosomes pass through the filter membrane to remove EVs with larger size, and then concentrated ultrafiltration is employed to retain exosomes on the filter membrane to remove small-molecule impurities [89]. Ultrafiltration is fast and easy to operate, but the membrane can clog, and the shear stress during filtration may damage the structure of exosomes [86]. To address these problem, tangential flow filtration (TFF) was developed. In TFF, the sample flows parallel to the membrane surface, which helps prevent clogging and improves processing performance [90].

#### 3.1.3. Size Exclusion Chromatography

SEC separates exosomes from soluble contaminants via porous polymer bead columns, and is highly effective at removing free proteins/lipoproteins frequently co-isolated with exosomes [91]. Compared with ultracentrifugation, SEC performs better in preserving exosome structure/biological activity and separating soluble impurities. However, the yield of SEC is still relatively limited [92]. Moleirinho et al. [93] developed a dual-column, semi-continuous SEC system combined with ultrafiltration, which significantly increased exosome yield.

#### 3.1.4. Polymer Precipitation

Polymer precipitation mainly separates exosomes based on their hydrophilic surface property and hydration layer stability. Polymer precipitation works by adding highly hydrophilic polymers to the sample. These polymers bind water molecules around exosomes and create a less soluble environment that induces the precipitation of exosomes [94] (Figure 3B). This method is widely used because it is simple, fast, inexpensive, and reproducible. And it is ideal for handling large sample volumes with less specialized equipment than ultracentrifugation [95]. Based on these advantages, most commercial exosome isolation kits are based on polymer precipitation [96,97]. However, although this approach generally yields a higher number of exosomes, the purity is lower than that achieved by ultracentrifugation [98], as other proteins and particles can co-precipitate. In addition, residual polymers may interfere with further analyses and affect experimental results [99,100]. To improve the compatibility with subsequent applications, polymer precipitation is often combined with other isolation methods. For example, Martínez-Greene et al. [101] combined polymer precipitation with size exclusion chromatography to efficiently isolate exosome subpopulations suitable for proteomic analysis.

### 3.2. Immunoaffinity-Based Isolation and Enrichment Methods

Physical methods allow convenient sample pretreatment, but as discussed above, they still lack specificity and cannot distinguish between different exosome subpopulations or non-exosomal particles with similar physical properties. As a result, immunoaffinity-based isolation and enrichment strategies have gained attention, as they enable more targeted and selective purification of exosomes.

Immunoaffinity methods isolate and purify exosomes based on specific interactions between exosome surface proteins (like CD63, CD9, CD81, and epithelial cell adhesion molecule (EpCAM)) and their corresponding antibodies [102]. In this technique, antibodies that recognize exosome markers are first attached to a solid surface, such as magnetic beads or culture plates. Then, the sample containing the target exosomes is added, allowing the exosomes to bind to the surface. Subsequently, specific elution methods are used to release exosomes from the surface, generating highly pure and specific exosome fractions [103] (Figure 3C). Thus, immunoaffinity isolation achieves the highest purity and can target specific subtypes of exosomes [104,105]. For example, Tauro et al. used a colon cancer cell line (LIM1863) to compare the performance of anti-EpCAM magnetic bead-based immunoaffinity isolation with ultracentrifugation and density gradient methods. They found that immunoaffinity isolation gave the highest exosome purity and increased the detection sensitivity of tumor-specific markers by 3–5-fold [106]. However, immunoaffinity methods achieve high purity at the cost of higher expense, greater time consumption and lower exosome yields [107]. In addition, traditional elution methods may damage exosomal membranes and impair their biological function. To solve this, Brambilla et al. [108] developed short peptides that bound specifically to antibodies and released exosomes without damaging their structure, which improved the immunoaffinity approach.

### 3.3. Microfluidics-Based Isolation and Enrichment Methods

Although conventional exosome isolation methods are widely used, they pose risks of exosome damage, low separation purity, and lengthy processing times, making them inadequate for growing research demands. In recent years, various novel exosome isolation technologies have been proposed and rapidly developed [102]. Microfluidic platforms have emerged as a compelling alternative to conventional bulk isolation methods, offering precise fluid control, reduced sample volume requirements, shorter processing times, and compatibility with downstream on-chip analysis.

Size-based passive microfluidic approaches exploit the physical dimensions of exosomes for label-free separation. Nanoporous membrane-integrated chips, such as the Exosome Total Isolation Chip (ExoTIC), used sequential filtration to remove large debris while retaining exosomes, achieving yields 4- to 1000-fold higher than ultracentrifugation from sample volumes as small as 10–100 μL [109]. Deterministic lateral displacement (DLD), which uses periodic nanopillar arrays to deflect particles along size-dependent trajectories, has been extended to the nanoscale regime and demonstrated separation of exosomes from colloids down to 20 nm [110]. More recently, DLD arrays combined with dielectrophoresis (DEP) have been reported to further improve separation resolution by exploiting both size and dielectric properties simultaneously [111].

Viscoelastic microfluidics represents another label-free strategy in which small amounts of biocompatible polymer additives induce elastic lift forces that scale with particle volume, pushing larger vesicles toward channel centerlines while allowing smaller exosomes to migrate to the sidewalls. This approach has achieved exosome purity above 90% and recovery rates above 80% from serum and cell culture media [80]. A further advance demonstrated direct isolation of small extracellular vesicles from whole blood using viscoelastic flow without any labeling steps, with 97% purity and 87% recovery [112].

Acoustofluidic separation applies surface acoustic waves generated by interdigitated transducer electrodes to create standing acoustic fields within microchannels. Particles experience differential acoustic radiation forces depending on their size, density, and compressibility, enabling continuous and label-free sorting. Acoustofluidic devices have been shown to isolate exosomes from whole blood with a blood cell removal rate of over 99.999% by integrating a cell removal module with a downstream vesicle-separation module [94] (Figure 3D).

Immunoaffinity-based microfluidic chips sacrifice label-free simplicity in exchange for subpopulation specificity. Channel surfaces or embedded beads functionalized with antibodies against tetraspanins (CD63, CD9, CD81) or disease-specific markers allow selective capture of defined exosome subpopulations. Herringbone micromixer structures increase the contact frequency between exosomes and functionalized surfaces, and have demonstrated more than 80% capture efficiency for tumor-derived exosomes from plasma, while standard UC isolation only recovered 15.5 ± 1.7% [113]. Immunomagnetic bead-based microfluidic devices have been applied directly to clinical blood samples, achieving sensitive and specific detection of EpCAM-positive exosomes from breast cancer patients [114].

## 4. Direct Detection of Intact Exosomes

The direct detection of intact-exosomes offers several advantages. First of all, this strategy eliminates the need for exosome lysis and protein extraction, simplifies the analysis process, reduces sample loss, and preserves the natural conformational integrity of membrane proteins. Secondly, intact exosome detection retains particle-level information. It can simultaneously quantify the absolute particle concentration, surface protein expression profiles, and the co-expression of multiple markers on the same vesicle. This co-expression information has special diagnostic value because tumor-derived exosomes are often distinguished not by the presence of a single biomarker, but by the co-occurrence of specific marker combinations on individual particles.

FET biosensors enable efficient direct detection of intact exosomes. The key to this method is to functionalize the FET sensing surface with antibodies or aptamers that specifically bind to exosomal surface markers. The detection works by taking advantage of the electrical properties of exosomes. Exosomes typically have a negative surface charge, with zeta potentials ranging from −10 to −30 mV. When exosomes bind to the recognition elements on the sensor, they cause measurable changes in the FET’s electrical characteristics, such as drain current, threshold voltage, and the shift of the Dirac point for graphene FETs, which could represent both qualitative and quantitative information about the exosomes. Table 1 lists the recent applications of FET biosensors in intact-exosome detection. Two major categories of surface biomarkers have been extensively exploited: one is the universal markers for exosomes like conserved tetraspanin proteins, the other is disease-related biomarkers. The strategic selection between these two categories enables FET biosensors to be optimized either for broad exosome capture or disease-specific diagnostic applications, thereby expanding their utility across both fundamental research and clinical settings.

### 4.1. Exosome Detection Based on Tetraspanins

The tetraspanin family, including CD63, CD9, and CD81, play important roles in processes such as cell adhesion, migration, signal transduction, and membrane fusion. These proteins are widely expressed on the surface of exosomes released from almost all cell types [133]. Because they are considered universal markers of exosomal membranes, tetraspanins are commonly used as key targets in FET biosensor-based exosome detection. By specifically recognizing one or more tetraspanins on the exosome surface, these biosensors can achieve efficient exosome capture and quantitative analysis.

**Figure 4:**
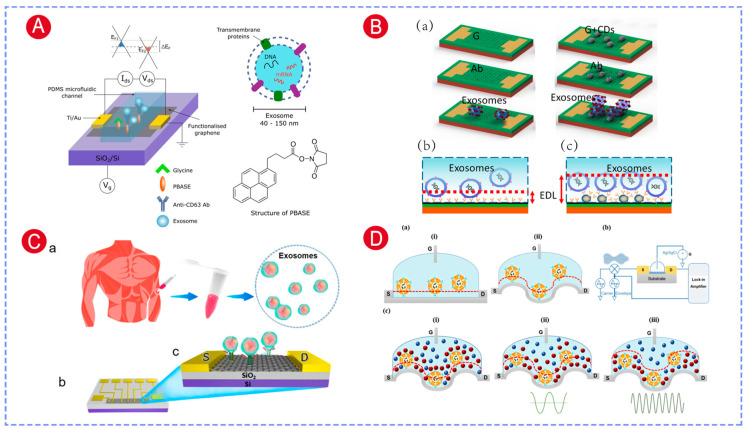
(**A**) Schematic of the functionalized biosensor with microfluidic integration. Schematic of the basic structure of an exosome and molecular structure of 1-pyrenebutyric acid N-hydroxysuccinimide ester (PBASE) [118]. Copyright (2019) Springer Nature. (**B**) Schematic illustration of the GFET and CDs-GFET sensors. (**a**) Device fabrication, surface modification, and capture of exosomes. (**b**,**c**) Schematic illustrations of the exosomes captured on (**b**) graphene and (**c**) CD-modified graphene [117]. Copyright (2021) American Chemical Society. (**C**) Schematic diagram of a CD63 antibody-functionalized RGO FET biosensor for detection of exosomes. (**a**) Exosomes are isolated and purified from the blood of patients. (**b**) RGO FET biosensor. (**c**) After anti-CD63 functionalization in the sensing region, exosomes can be directly bound to the CD63 antibody functionalized RGO FET biosensor for electrical and label-free detection [23]. Copyright (2019) American Chemical Society. (**D**) (**a**) Debye length modulation in (**i**) flat graphene and (**ii**) deformed graphene, (**b**) circuit of high-frequency operation in heterodyne mode, (**c**) Impact of AC electric field carrier frequency on the formation of electrical double layers: (**i**) when dc electric field is applied, (**ii**) when a small carrier frequency is applied, and (**iii**) when a high carrier frequency is applied [27]. Copyright (2025) American Chemical Society.

Graphene is an ideal channel material for constructing highly sensitive FET biosensors due to its two-dimensional structure, ultrahigh carrier mobility, excellent electrical conductivity, and favorable biocompatibility. Single-layer chemical vapor deposition (CVD) graphene or reduced graphene oxide (rGO) is often used as the conductive channel in graphene-based FET (GFET) devices. Tsang et al. developed a chemically functionalized GFET sensor for label-free exosome detection. In this device, CVD-grown monolayer graphene served as the transistor channel, while 1-pyrenebutyric acid N-hydroxysuccinimide ester (PBASE) was used as a linker to immobilize anti-CD63 antibodies. When integrated with microfluidic technology, the platform enabled highly specific exosome detection at concentrations as low as 0.1 μg/mL (Figure 4A) [118]. Similarly, Hajian et al. fabricated monolayer GFET chips via CVD graphene and introduced an exosome detection platform (EV-chip) coupled with a portable FET readout device. In this system, phenylboronic acid (PBA) was employed as the chemical linker for CD63 antibody immobilization, achieving a detection limit of 2 × 10^4^ particles/mL. Furthermore, detection of CD151-positive exosomes showed the potential diagnostic value of CD151 in aging and age-related diseases [32].

To overcome the charge screening of conventional GFET sensors, Ramadan et al. introduced a carbon dot (CD)-based enhancement strategy. CDs with a diameter of 5–10 nm were deposited onto the graphene surface by drop-casting. These CDs played two key roles. First, the spherical nanostructure formed a 3D sensing interface, which in turn increased the loading density of CD63 antibodies and enhanced exosome capture. Second, the CDs modified the electrical double-layer capacitance of graphene and effectively extended the Debye length, thereby suppressing the charge screening. Together, these effects enabled exosome binding-induced charge changes to convert more efficiently into clear Dirac voltage shifts, greatly enhancing sensor sensitivity. As a result, the detection limit was reduced to 100 particles/μL, a three-orders-of-magnitude improvement over the unmodified GFET devices (Figure 4B) [117]. To further improve sensitivity, our group developed an rGO-FET biosensor. RGO was deposited onto the FET channel by drop-casting, and the surface was subsequently functionalized with CD63 antibodies [23]. This platform enabled label-free and highly sensitive exosome quantification (Figure 4C). And the clinical sample analysis results revealed that CD63-based serum exosome levels differed significantly between prostate cancer patients and healthy subjects (*p* < 0.01).

Besides material modification, structure engineering of graphene is another effective way to overcome the Debye screening effect. Mukherjee et al. [27] designed a wrinkled GFET for enhanced exosome detection. To fabricate the wrinkled graphene on a glass substrate, thermally reduced GO was deposited onto pre-assembled silver nanoparticles. And the surface was subsequently functionalized with PBASE and CD91 antibodies. This work also used a dual strategy that combined the “wrinkled structure” with “AC heterodyne mode operation”, both reducing the Debye screening effect and significantly enhancing the detection sensitivity of exosomes. With this approach, the sensor achieved stable detection of lung cancer cell-derived exosomes in human serum at a low LOD of 1200 exosomes/mL (Figure 4D). The entire detection process took merely 25 min, making this a rapid, highly sensitive method with great promise for early cancer screening.

Efficient exosome isolation is critical for the accuracy and sensitivity of detection results. Kim et al. prepared a conductive helical microfluidic channel via uniform mixing of silver powder with PDMS and subsequent molding into a pre-patterned structure [124]. The helical channel design generates hydrodynamic forces, while an applied voltage produces additional dielectrophoretic (DEP) forces. The combination of these two forces enables rapid and highly efficient exosome separation in a simple manner. Furthermore, the separated exosomes were detected using an rGO-FET sensor, and the signal intensity was about four times higher than that of the supernatant before separation.

**Figure 5:**
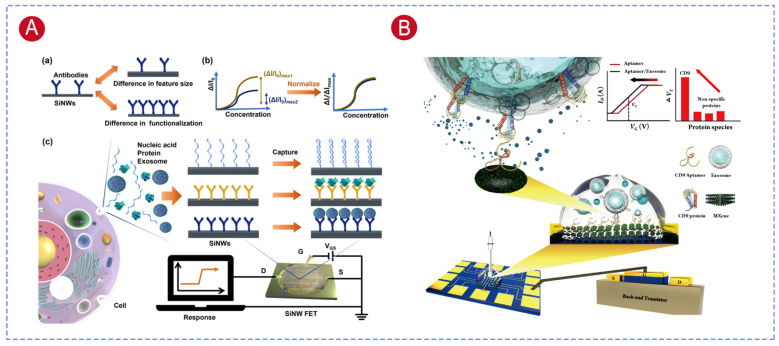
(**A**) Calibration strategy of SiNW FET biosensors and its applications in biosensing. (**a**) Issues in manufacturing and functionalization of SiNW FET biosensor. (**b**) Calibration schematic diagram of SiNW FET biosensor. (**c**) Applications of SiNW FET biosensors in biosensing [61]. Copyright (2024) American Chemical Society. (**B**) Schematic diagram of exosome detection using the EGFET aptasensor [119]. Copyright (2023) American Chemical Society.

In addition to graphene, silicon nanowire (SiNW) FETs are also commonly employed for exosome detection. However, differences in nanowire dimensions and surface functionalization can cause large variations in threshold voltage shifts when detecting the same exosome concentration. This variability makes it difficult to reliably compare exosomal membrane protein expression levels across different sensors. To overcome this challenge, Chen et al. proposed a saturation response calibration strategy. By combining the sensing principle of SiNW FETs with the Langmuir–Freundlich model, detection response (ΔI/I_0_) was normalized using the saturation response (ΔI/I_0_)max of the sensor. This strategy enabled ultrasensitive, rapid, and label-free detection of exosomes and provided a practical pathway for translating SiNW FET biosensors from theoretical research to practical sensing applications (Figure 5A) [61].

Extended-gate (EG-FET) sensors offer the advantage of rapid replacement of the sensing membrane, which avoids signal drift caused by repeated cleaning and makes EG-FETs well-suited for clinical testing [134]. An et al. incorporated MXene nanomaterials into the sensing membrane of an EG-FET. The sheet-like structure of MXene increases the specific surface area of the sensing membrane, thereby enhancing exosome capture efficiency. Also, its high electrical conductivity amplifies the charge variations induced by exosome binding. A highly specific CD9 aptamer was employed as the recognition receptor to target CD9 proteins on the exosome surface. This sensing platform exhibited excellent detection performance with a linear detection range of 1 × 10^3^–1 × 10^7^ particles/mL and a detection limit of 6.41 × 10^2^ particles/mL (Figure 5B) [119].

OECT-based bioelectronic systems possess superior volumetric signal amplification and excellent compatibility with aqueous biological environments, making them ideal for high-throughput quality monitoring in EV industrial production. Frusconi et al. developed a 3 × 4 matrix OECT sensing array with PEDOT:PSS as the conductive channel material, and modified the channel using azide-functionalized poly-L-lysine (PLL-N_3_) to support strain-promoted alkyne–azide cycloaddition (SPAAC) click chemistry [67]. The covalent interaction between azide groups on the channel and dibenzocyclooctyne (DBCO) labeled on EV membrane protein amino groups achieves marker-independent pan-specific EV capture, which eliminates quantification bias caused by heterogeneous expression of EV surface proteins. Integrated with 3D-printed fluidic components and a portable silicon-based electronic reader, this disposable cartridge system supports 12-channel parallel detection. The biosensing platform completes the entire test in only 15 min, reaching a limit of identification (LoI) of 6 × 10^6^ particles/mL, with both false-positive and false-negative rates kept below 1%. It provides highly reliable total EV quantification for practical use in biomanufacturing and pharmaceutical production processes.

Compared with detection of a single tetraspanin protein, simultaneous analysis of multiple tetraspanins on exosomes enables more precise exosome subtyping. Qin et al. developed a SiNW Bio-FET biosensor for highly sensitive exosome detection. SiNW channels were fabricated using an etching process, followed by channel doping through BF_2_^+^ ion implantation at a dose of 1 × 10^13^. The SiNWs were then functionalized with APTES and glutaraldehyde to immobilize CD81 antibodies, resulting in a low limit of detection of 1078 particles/mL. Furthermore, SiNW Bio-FETs modified with CD9, CD81, and CD63 antibodies were used to distinguish exosomes derived from human promyelocytic leukemia (HL-60) cells under three different conditions: control group, lipopolysaccharide (LPS)-induced inflammation group, and LPS-plus-romidepsin (FK228) drug treatment group [74].

In conclusion, although tetraspanins are most commonly used for the quantitative detection of exosomes, they have also been explored as diagnostic markers in various diseases, largely due to their differential expression on the membranes of exosomes originating from distinct cell types. However, because tetraspanins are expressed on exosomes derived from both healthy and diseased cells, approaches based on these markers lack disease specificity and are therefore unable to reliably discriminate between exosomes associated with different diseases or distinct pathological subtypes. Therefore, the next section focuses on disease-specific biomarkers, exploring their application in FET-based exosome detection to achieve more precise and targeted disease diagnosis.

### 4.2. Exosome Detection Based on Specific Disease Biomarkers and Its Application in Disease Diagnosis

Proteins on the exosome membrane surface carry the pathological characteristics of their parental cells. Protected by the vesicle membrane, these proteins exhibit high stability and can be non-invasively obtained from body fluids, making them highly promising disease biomarkers [135]. In particular, the detection of tumor-specific protein markers on intact exosome membranes enables precise identification of tumor-derived exosomes and provides a reliable non-invasive approach for early cancer diagnosis and pathological classification.

For the diagnosis of hepatocellular carcinoma (HCC), our group developed a dual-aptamer RGO-FET sensor by in situ reduction and growth of gold nanoparticles (AuNPs) on RGO, accompanied by the simultaneous immobilization of thiolated TLS11a and EpCAM aptamers. This platform enabled the detection of HepG2 cell-derived exosomes. The dual-aptamer detection strategy showed a significant signal enhancement, achieving high sensitivity and specificity with a LOD of 84,000 particles/mL. Using this platform, significant differences in HepG2-derived exosome levels were found in clinical blood samples from HCC patients and healthy individuals [22].

**Figure 6:**
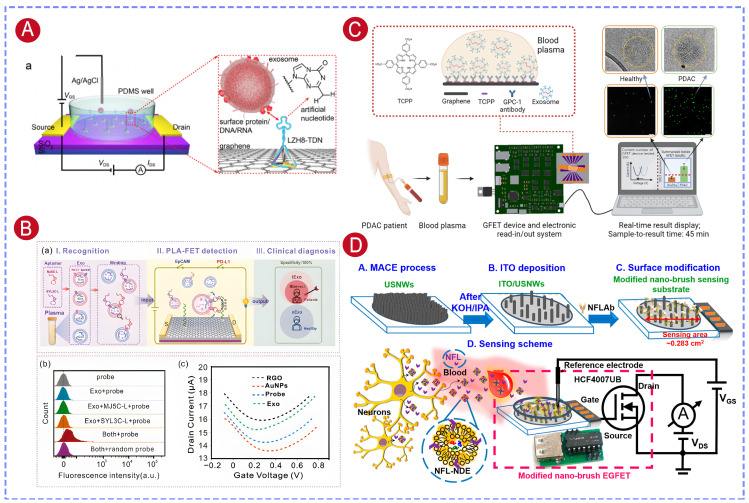
(**A**) Schematic diagram of an AN-Apta-FET biosensor with AN (P). Inset: LZH8-TDNs with artificial nucleotides on graphene specifically recognize exosomes, altering I_DS_ signals [120]. Copyright (2023) American Chemical Society. (**B**) Schematic diagram and Feasibility. (**a**) Schematic of PLA-FET sensor for detection of tExo-PD-L1. (**b**) Flow cytometry validation of PLA binding between MJ5CL and SYL3C-L aptamers and connector probes. (**c**) Incremental Id-Vg curves for the PLA-FET biosensor during AuNP deposition, connector probe modification, and A375 exosome detection processes [26]. Copyright (2025) Elsevier. (**C**) Schematics of detection of PDAC exosomes using GFETs with portable electronics and real-time detection results [125]. Copyright (2023) American Chemical Society (**D**) Schematic diagram of the integrated nano-brush sensing substrate with the EGFET device [122]. Copyright (2023) American Chemical Society.

However, natural nucleotide aptamers have inherent structural limitations and poor conformational diversity, which narrow the range of analytes detectable by aptamer-based biosensors. To overcome these limitations, artificial nucleotide aptamers have been developed. These aptamers are derived from natural nucleotides through chemical modifications that introduce non-natural bases, backbone structures, or side-chain functionalities. As a result, the specific target-binding capability is preserved and the structural diversity and application scope are expanded. Chen et al. constructed a GACTZP-DNA library containing Z/P artificial nucleotides with a 25-nucleotide random region. High-affinity sequences were enriched through co-incubation with HepG2 cells, followed by negative selection against normal hepatocytes to eliminate non-specific binders. After 13 rounds of PCR amplification and fluorescence-based validation, the LZH8 aptamer, which specifically targeted HepG2 cell-derived exosomes, was successfully identified. To realize ultrasensitive detection, the graphene surface was sequentially modified with 1-pyrenebutyric acid N-hydroxysuccinimide ester (PASE) and tetrahedral DNA nanostructures, into which the LZH8 aptamer was incorporated. This strategy achieved an LOD as low as 242 particles/mL and could discriminate serum samples from HCC patients and healthy individuals within 9 min, verifying its strong potential for rapid clinical diagnostics (Figure 6A) [120].

For prostate cancer diagnosis, Choi et al. [30] developed an explainable artificial intelligence (XAI)-based screening system, which used logistic regression alongside kernel SHAP analysis to interpret feature importance. Urinary exosomal biomarker signals (TMEM256, flotillin-2, and PSMA) detected by dual-gate FET (DGFETs) biosensors were integrated with PI-RADS scores in this system. In the blind testing of 102 samples, the system achieved an AUC value of 0.93, and its diagnostic accuracy for PI-RADS 3 lesions was improved from 30.4% to 66.7%. The TMEM256 was also recognized as a key biomarker. Conclusively, this approach provided clinicians with an interpretable, non-invasive, and efficient diagnostic tool.

To solve the issue of insufficient specificity of single-exosomal-membrane-protein biomarker-based cancer diagnosis, our group integrated a proximity ligation assay (PLA) with RGO-FETs to develop a dual-aptamer strategy (Figure 6B) [26]. Two aptamers, MJ5C-L targeting PD-L1 and SYL3C-L targeting EpCAM, were employed to construct an AND-logic gate sensor. The two aptamers only came into close proximity when exosomes co-expressed both markers, which induced a higher local concentration of extension sequences. As a result, the ligation probes were subsequently activated on the FET surface. The LOD for tumor-derived exosomal PD-L1 (tExo-PD-L1) was 52.48 fg/mL. Clinical sample analysis showed that tExo-PD-L1 detection distinguished lung cancer patients from healthy people more accurately than total Exo-PD-L1 detection.

For pancreatic cancer diagnosis, Yin et al. [125] developed an accurate, stable and portable GFET array biosensor platform. By detecting glypican-1 (GPC-1) expression on the membranes of plasma exosomes, this platform enabled the diagnosis of pancreatic ductal adenocarcinoma (PDAC) within 45 min (Figure 6C). An internal reference channel integrated into the GFET chip effectively suppressed background noise, which significantly reduced false-positive rates and simultaneously improved detection accuracy and measurement reproducibility. Analysis of samples from 18 PDAC patients and 8 healthy controls demonstrated that this GFET biosensor array reliably distinguished between the two groups and successfully detected early-stage pancreatic cancers, including stage I and II.

Detection of neuronal-derived exosomes in body fluids offers a promising liquid biopsy approach for the diagnosis of neurodegenerative diseases. Lin et al. [122] fabricated a “nanobrush” sensing interface by coating up-standing SiNWs with indium tin oxide (ITO) and subsequently functionalizing the surface with antibodies against neurofilament light chain (NFL). This nanobrush structure was connected to a MOSFET as an extended gate. By measuring serum NFL levels, this sensor was able to differentiate between different stages of Alzheimer’s disease (AD). Moreover, the platform was further applied to the detection of NFL-positive neuron-derived exosomes (NFL-NDEs (Figure 6D). Owing to the high surface area of the nanobrush architecture, target capture efficiency was significantly enhanced, which enabled label-free detection within 2 min and achieved an LOD as low as 60 particles/mL.

Conventional OECT-based EV detection depends on slow passive diffusion, leading to low sensitivity and lengthy testing processes. To address these practical issues, Li et al. developed a fully integrated sensing platform that combines acoustoelectric enrichment with OECTs [123]. They applied focused surface acoustic waves along with electric pulses to actively transport and densely concentrate EVs onto the transistor’s sensing zone, and fixed specific antibodies to capture AD-typical EVs loaded with Aβ and tau proteins. This integrated sensor gave a signal enhancement of more than 280-fold within 30 s, reached an ultra-low detection limit of 500 EV particles per milliliter, and finished the full detection in just 2 min. Tests on clinical serum samples confirmed the platform could clearly distinguish AD patients from healthy people. In 5xFAD mouse models, it also effectively tracked AD progression, and its results showed a strong correlation with Aβ plaque burden measured by MRI.

## 5. Detection of Exosomal Lysates

While detecting intact exosomes gives important information about exosome concentration and surface phenotypes, analysis of exosomal contents can reveal much deeper molecular details about how a disease develops and its current state. Table 1 lists the recent applications of FET biosensors in exosomal lysate detection. To detect these internal components, exosomes must first be lysed, and FET biosensors can achieve highly sensitive detection of these components with proper surface functionalization.

### 5.1. Exosome Lysis Methods

Exosome lysis is an important step for releasing internal biomarkers for subsequent detection. This process needs to break down the exosomal lipid bilayer effectively, and at the same time, to preserve the integrity of internal biomolecules against degradation or denaturation. Lysis methods are generally classified into chemical and physical approaches.

Chemical lysis uses chemical reagents such as surfactants and lysis buffers to disrupt the lipid membranes of exosomes through hydrophobic interactions or ionic-bond disruption. For instance, surfactants like Triton X-100 insert into the lipid bilayer and weaken the hydrophobic interactions between phospholipids [24]. Lysis buffers commonly contain detergents as well as protease and RNase inhibitors. The detergents interact with membrane lipids to form micelles that destabilize the membrane, while the inhibitors help preserve proteins and microRNAs during the lysis process [28].

Physical lysis disrupts exosomal membranes through temperature changes or mechanical forces, primarily including freeze–thaw cycling and sonication. Freeze–thaw cycling consists of repeated freezing (typically 3–5 cycles) in liquid nitrogen or at −80 °C, followed by thawing at room temperature [25]. During this process, the formation and expansion of ice crystals generate mechanical stress that leads to membrane rupture. As a reagent-free approach, it better preserves the native state of biomarkers, although lysis may be incomplete. This approach is particularly suitable for applications requiring mild processing conditions. Sonication induces exosome lysis through cavitation effects and localized pressure generated by ultrasonic waves. Proper optimization of sonication power and exposure time are necessary to prevent protein denaturation and nucleic acid degradation.

### 5.2. MicroRNA Detection

Exosomal microRNAs have gained increasing attention as disease biomarkers in liquid biopsy due to their high stability and tissue specificity. Compared to the detection of surface proteins on exosomes, measuring the expression levels of specific intravesicular microRNAs provides a more accurate representation of disease-related molecular changes and disease progression. By functionalizing FET biosensors with nucleic acid probes, target microRNAs released after exosome lysis can be directly recognized and quantified, enabling rapid and highly sensitive detection for exosomal microRNA-based diagnosis. Huang et al. [28] immobilized thiol-modified cDNA probes on the gold gate electrode of a MOSFET device and achieved highly sensitive and specific detection of microRNA-195 and microRNA-126, two breast cancer-related biomarkers, from exosome lysates, with an LOD as low as 80 aM. In addition, a microfluidic chip was integrated onto the gate electrode, resulting in an extracellular vesicle capture efficiency of 84% within 4 h and separation efficiencies of 85% and 94% for microRNA-195 and microRNA-126, respectively, within 20 min. This integrated system enabled automated exosome isolation, microRNA separation, and electrical detection, showing its potential as a practical platform for precise and automated breast cancer diagnosis [29].

Indium gallium zinc oxide (IGZO) is widely used as a transistor channel material due to its high carrier mobility. However, IGZO thin-film transistors (TFTs) are highly sensitive to moisture, which can degrade carrier transport and limit long-term stability. Lv et al. reported an aluminum-induced crystallization (AIC) IGZO TFT biosensor for ultrasensitive miRNA detection. By introducing an Al layer onto the IGZO thin film and applying high-temperature treatment, partial crystallization of the top channel was induced, leading to the formation of covalent bonds between the IGZO channel and the Al layer. In addition, the Al layer formed an AlO_x_/Al/AlO_x_ floating-gate structure that effectively isolated the liquid environment from the IGZO channel, thereby reducing carrier scattering caused by moisture. At the same time, this structure allowed efficient transmission of weak bioelectrical signals through tunneling effects. As a result, the electrical performance and sensing sensitivity of the TFT were significantly improved, enabling the detection of gastric cancer-related microRNA-106a with a LOD of 1.65 fg/mL [25].

To reduce the environmental impact of conventional sensors and meet the requirements of disposable clinical testing, Yin et al. [31] developed an IGZO-TFT biosensor fabricated on a nanocellulose paper substrate. Specific DNA probes immobilized in the IGZO channel selectively hybridize with target miRNAs extracted from glioma-derived exosomes (g-miRNA), forming DNA-RNA duplexes. The accumulation of negative charges from the duplexes modulates the channel conductivity, leading to a positive shift in threshold voltage and a decrease in the source–drain current. A polyimide (PI) coating was introduced to provide sufficient surface smoothness for IGZO thin-film deposition, while the nanocellulose substrate could be fully degraded in alkaline solution, thereby reducing disposal concerns. This biosensor achieved an LOD of 350 aM for g-miRNA, demonstrating its potential for early glioma diagnosis.

**Figure 7:**
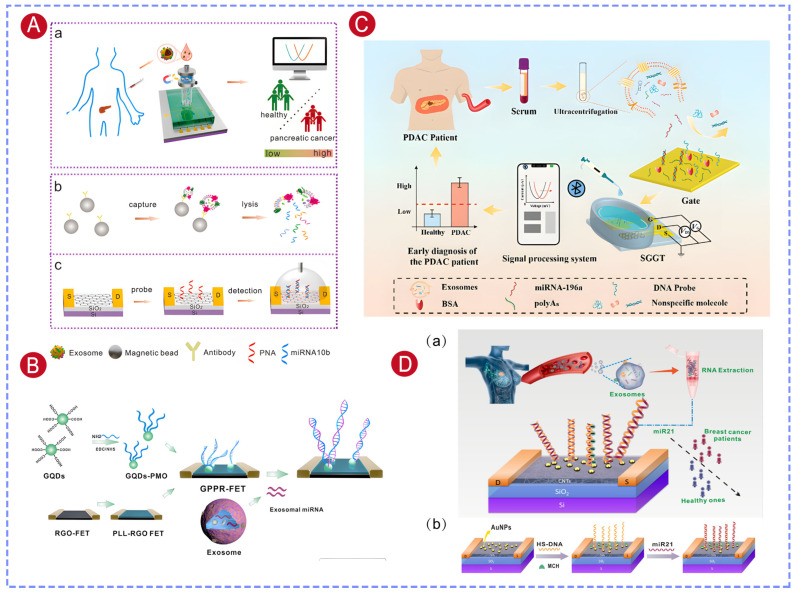
(**A**) Schematic diagram of the integrated microsystem for the detection of PC-derived exosomal miRNA10b. (**a**) A schematic diagram of the integrated microsystem to detect exosomal miRNA in the blood. (**b**) Dual antibody functionalized magnetic beads capture the exosomes and subsequently lyse them to release miRNA. (**c**) PNA-functionalized FET biosensor to detect miRNA10b [128]. Copyright (2023). Elsevier. (**B**) Schematic principle of GQD-PMO-functionalized PLL-RGO-FET (GPPR-FET) for detection of exosomal miRNA [136]. Copyright (2022). Cell Press. (**C**) Schematic diagrams of the detection process of miRNA-196a in exosomes through the SGGT sensor [131]. Copyright (2025). Wiley. (**D**) (**a**) Schematic diagram of ultrasensitive detection of exosomal miR21 using the DNA-functionalized CNT FET biosensor. (**b**) Schematic illustration of stepwise DNA probe immobilization and DNA-miRNA hybridization of the CNT FET sensor [129]. Copyright (2021). American Chemical Society.

GFETs have demonstrated outstanding performance in detecting microRNAs from exosome lysates. In our work, peptide nucleic acid (PNA) probes were immobilized on the RGO-FET channel to enable selective and sensitive detection of exosomal miRNA-10b. In addition, magnetic beads functionalized with dual antibodies (GPC-1 and EpCAM) were used for exosome capture. Compared with single-antibody-modified beads, the dual-antibody strategy more efficiently captured exosomes derived from the pancreatic cancer cell line PANC-1, allowing effective discrimination from exosomes originating from MCF7, MCF10A, and H6C7 cells. The sensor achieved an LOD of 78 fM for pancreatic cancer exosomal miRNA-10b (Figure 7A) [128]. Furthermore, analysis of clinical samples demonstrated that this method could reliably distinguish pancreatic cancer patients from healthy individuals.

To further enhance miRNA detection sensitivity, Li et al. developed an RGO-FET sensor functionalized with graphene quantum dot–phosphorodiamidate morpholino oligomer (GQDs-PMO). In this design, the RGO surface was first modified with poly-L-lysine (PLL), after which the GQDs-PMO conjugates were deposited onto the channel by drop-casting. GQDs not only had a high specific surface area that elevated PMO probe immobilization density, but also had good electrical conductivity that improved signal transduction efficiency. As a result, the sensor achieved an extremely low LOD of 85 aM for breast cancer-associated exosomal miRNA-21 (Figure 7B) [130,136].

Non-specific adsorption in complex biological fluid environments remains a significant challenge for accurate detection of low-concentration miRNAs. To solve this, Song et al. developed an antifouling solution-gated graphene transistor (SGGT) sensor. The gold gate electrode was functionalized with ssDNA probes specific to miRNA-196a, then blocked with bovine serum albumin (BSA) and modified with polyA_8_. The polyA_8_ layer made the surface more hydrophilic and formed a dense hydration layer, which effectively suppressed the non-specific adsorption of hydrophobic contaminants. This significantly reduced background noise in complex biological settings, and thus improved the sensor’s detection specificity and sensitivity. The sensor achieved an LOD of 0.18 aM for miRNA-196a, and its successful application in clinical exosome analysis enabled reliable differentiation of PDAC patients from non-PDAC individuals (Figure 7C) [131].

Semiconducting carbon nanotubes (CNTs), owing to their excellent electron transport properties, high mechanical strength, and good biocompatibility, have also been widely explored in FET biosensors. In our work, polymer-sorted, high-purity semiconducting CNT thin films were used as the channel, and a floating-gate structure based on a Y_2_O_3_ insulating layer was introduced [129]. The Y_2_O_3_ layer effectively isolated the liquid environment from the CNT channel, while gold nanoparticles deposited on the Y_2_O_3_ surface provided sites for probe immobilization, thereby enhancing target binding efficiency (Figure 7D). This device architecture enabled label-free detection of the breast cancer biomarker miRNA-21, achieving an LOD as low as 0.87 aM. Furthermore, the sensor was successfully applied to the analysis of exosomal miRNA-21 from clinical samples and demonstrated reliable discrimination between breast cancer patients and healthy individuals.

### 5.3. Protein Detection

**Figure 8:**
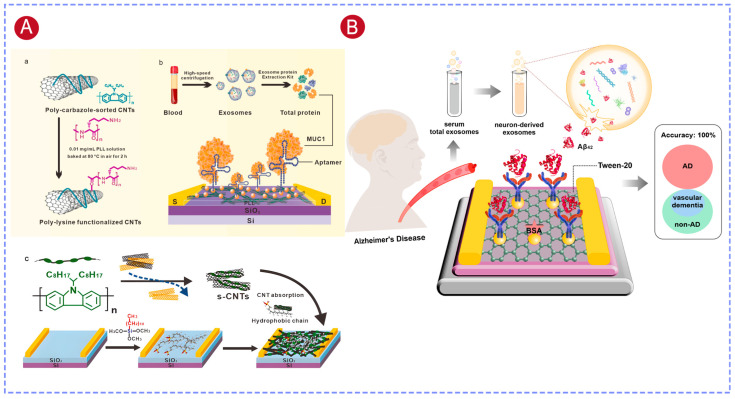
(**A**) (**a**) Schematic diagram of functionalization of CNTs with PLL under mild conditions (**b**) Schematic diagram of the aptamer-functionalized CNT FET biosensor for detection of cancer-derived exosomal MUC1. (**c**) Schematic illustration of stepwise CNTs deposition on the CNT FET sensor [24]. Copyright (2023). Elsevier. (**B**) Schematic illustration of the G-EGT biosensor for detection of NDE-A*β*42 in Alzheimer’s disease patients [126]. Copyright (2023). American Chemical Society.

Exosomal proteins are closely linked to the initiation and progression of a wide range of diseases, including cancer, cardiovascular disorders, and neurodegenerative diseases, and therefore represent important biomarkers for liquid biopsy. Exosomal MUC1 protein has been identified as a promising marker for early detection of breast cancer. In our work, a CNT-FET sensor was constructed by sequentially modifying the CNT surface with poly-L-lysine (PLL), gold nanoparticles (AuNPs), and thiolated aptamer probes, enabling label-free detection of MUC1. The introduction of AuNPs increased the effective surface area of the sensing interface, enhanced the immobilization density of aptamer probes, and improved signal transduction upon target binding. The sensor achieved a detection limit of 0.34 fg/mL, and analysis of clinical samples demonstrated its capability to reliably distinguish breast cancer patients from healthy individuals (Figure 8A) [24].

Neuron-derived exosomal Aβ42 (NDE-Aβ42) can cross the blood–brain barrier and is highly correlated with the pathological progression of AD. Our group [126] developed a G-EGT-based nanosensor for detecting NDE-Aβ42 levels in serum. The sensor employed AuNP modification to enhance antibody probe loading capacity, and a dual-blocking strategy using BSA and Tween-20 to minimize non-specific adsorption, achieving ultrasensitive and highly specific detection of Aβ42 with a detection limit as low as 447 ag/mL (Figure 8B). Clinical sample analysis demonstrated that this sensor achieved 100% accuracy in distinguishing AD patients from non-AD individuals, providing a reliable detection approach for early diagnosis of AD.

Additionally, our group developed a CNT-FET sensor by modifying the CNT surface with a polydopamine (PDA) film to immobilize antibodies against α-synuclein (α-Syn) and phosphorylated α-Syn (pS129-α-Syn), enabling simultaneous detection of dual Parkinson’s disease (PD) biomarkers [127]. Exosome-carried α-Syn oligomers could cross the blood–brain barrier and more accurately reflect the pathological state in the brain, while pS129-α-Syn served as an indicator of disease progression, with increasing plasma levels observed as Parkinson’s disease advances. Combining these two biomarkers for detection provides higher diagnostic accuracy than single-biomarker analysis. The sensor had a LOD of 9.21 fg/mL for α-Syn oligomers and 2.67 fg/mL for pS129-α-Syn, respectively, improving the sensitivity by 10–100-fold compared with conventional electrochemical methods. Analysis of clinical samples proved that simultaneous detection of exosomal α-Syn and plasma pS129-α-Syn increased the diagnostic accuracy to 97% for PD.

Blood EV-carried Aβ and tau proteins stand out as stable and representative biomarkers for AD, but relying on just one marker rarely gives enough confidence for clinical diagnosis. Zheng et al. built a label-free bioelectronic platform that combines OECTs with microelectrode arrays (MEAs), allowing simultaneous detection of four key AD biomarkers in blood EVs: Aβ_1–40_, Aβ_1–42_, total tau, and phosphorylated tau [132]. Using a specially designed hybrid self-assembled monolayer interface, the system supports high-density antibody attachment and strong resistance to non-specific fouling, so it can detect target molecules down to the zeptomolar level, with nearly single-molecule sensitivity. The whole multiplex test takes only 20 min, and in clinical trials with 40 samples, the platform correctly identified every case with 100% accuracy by combining signals from multiple biomarkers, clearly outperforming single-marker methods. After being validated with real patient serum samples, this multiplexed OECT-based approach offers a highly reliable, minimally invasive way to accurately diagnose AD and track how the disease develops over time.

Lastly, the two detection strategies based on the intact exosome and exosome lysis surveyed in Section 4 and Section 5 differ in signal origin, sensitivity, and clinical role, as summarized in Table 2.

Signal origin. As established in Section 2.2, the two strategies probe charges at very different length scales relative to λ_D_. In intact-exosome detection, only membrane charges within λD of the channel contribute effectively to ΔV_g_^eff^; and EDL restructuring by the bulky particle also contributes as concurrent transduction pathway. In lysate-based detection, miRNA or protein targets bind directly within a few nanometers of the channel, and electrostatic gating dominates as in classical small-molecule FET assays.

Sensitivity. Intact-exosome assays typically achieve 60–10^5^ particles/mL and lysate-based assays routinely reach the attomolar range for nucleic acids and the attogram-to-femtogram range for proteins.

Clinical information and applications. Intact-exosome detection preserves particle-level information, like vesicle counts and marker co-expression on the same vesicle, which is important for identifying tumor-derived exosome subpopulations. Lysate-based detection sacrifices this information in exchange for sensitive molecular quantification, making it well suited for early diagnosis and treatment monitoring. The two approaches are therefore complementary, and on-chip integration of immunocapture, lysis, and cargo quantification represents a promising direction for next-generation FET liquid-biopsy platforms.

## 6. Challenges and Future Perspectives

FET biosensors show great potential for exosome detection, but moving this technology from lab research to commercial use still comes with many challenges. Below, we summarize the major challenges and point out the development directions that may accelerate translation from proof-of-concept to real diagnostic platforms.

One of the most fundamental physicochemical limitations of FET biosensors is the Debye screening effect [137]. As discussed earlier, under physiological salt concentrations, the Debye length is only approximately 0.7 nm, far smaller than typical capture probes. This problem is particularly distinct for intact-exosome detection, considering the relatively large size of exosomes compared to the Debye length. As a result, charge perturbations associated with exosome binding are largely screened in electrolyte, leading to a markedly attenuated transduction signal and, consequently, degraded analytical performance of FET-based exosome assays [73,75]. To address this problem, we can either shorten the effective sensing distance or increase the local Debye length. The most straightforward approach is buffer dilution. By reducing ionic strength to 0.001× PBS, the Debye length can be extended to approximately 24 nm, which is sufficient to cover the antibody-exosome binding interface. The second approach is the engineering of the channel material interfaces. Constructing 3D nanostructures at the sensing interface can modulate the ion distribution and increase the Debye length [138]. Additionally, surface modification with polymers or lipid layers can tune interfacial dielectric properties, block ionic contact, and then effectively enlarge the Debye length [139]. The third approach is the optimization of biorecognition elements. This includes using the Fab or F(ab′)_2_ fragments of antibodies, nanobodies, aptamers and nano-molecular imprinting polymers (nMIPs) to reduce the probe size in order to shorten the distance from the target to the sensing surface [140,141]. In addition, enzymatic amplification can translate the macromolecular recognition into the local accumulation of small charged products within the Debye length [142]. Lastly, the external field perturbation is employed to disrupt the electrical double layer and avoid ionic screening [143]. These strategies effectively suppress the Debye screening effect in high-ionic-strength solutions, therefore enhancing the sensitivity, detection range, and stability of FET sensors.

Secondly, the quantitative accuracy of FET-based exosome detection is not solely determined by exosome concentration. FET signals are also influenced by the physical and electrical properties of individual exosomes, including particle size, membrane surface charge, and surface marker density. These properties vary widely across individuals, disease states, and even subpopulations within the same clinical sample, meaning that exosomes at the same concentration can produce signals that differ by orders of magnitude. This makes it difficult to establish a reliable calibration curve for clinical samples and limits reproducibility across different laboratories [144]. A practical strategy is to pre-enrich specific exosome subpopulations using immunoaffinity-based isolation before FET measurement, which reduces sample heterogeneity and improves signal consistency [145]. In the longer term, combining FET with complementary detection methods may allow simultaneous measurement of both electrical and physical parameters, providing a more reliable basis for quantification [146]. Further promising approaches include developing multi-channel FET arrays capable of simultaneously detecting multiple surface markers, enabling comprehensive characterization of exosome populations through multiparameter analysis, and integrating machine learning algorithms to analyze signals from multiple sensors and establish more accurate disease diagnostic models.

Thirdly, current FET sensors for exosome detection still face several challenges for real application. These include low integration levels, poor working efficiency caused by the disconnection between sample processing and detection steps, and limited accessibility due to reliance on sophisticated instruments. Future progress will rely on tighter integration of FET sensors with microfluidic systems. Such integration allows exosome enrichment and detection to be performed on a single chip. By combining multiple enrichment strategies on-chip, automated isolation of exosomes from complex biological samples can be achieved. In addition, aligning FET sensing arrays directly with microfluidic channels enables in situ detection. This eliminates sample transfer losses and builds toward a more integrated and multifunctional exosome analysis platform.

Beyond functional integration, miniaturization and portability are key next steps. Incorporating low-power readout circuits, compact power modules, and onboard data processing units into the system can reduce reliance on bulky laboratory instruments. This would allow high-performance exosome testing to move from central laboratories into POCT applications. Adding wireless communication modules to connect with smartphones would further support real-time data processing, result display, and remote data sharing. Together, these improvements would expand the use of FET-based detection in resource-limited settings such as primary care clinics and at the bedside, and bring the technology closer to real clinical use.

## Figures and Tables

**Table 1:** FET biosensors for exosome detection.

	Target Analyte	Device Architecture	Sensor Fabrication	Linear Range	Detection Limit	Clinical Sample Detectability	Ref.
intact exosome	CD151	Liquid-gate FETs	Graphene/PBA/antibody(channel)Ti/Pt(gate)	10^5^–5 × 10^9^ particles/mL	2 × 10^4^ particles/mL	Effectively distinguish between healthy individuals and the early diagnosis of age-related diseases	[32]
	biotin-modified exosome	Liquid-gate FETs	RGO/PASE/SA (channel) Ag/AgCl(gate)	10^4^–10^9^ particles/mL	20 particles/μL		[115]
	CD63	Back-gate FETs	SiNW/APTES/GA/antibody (channel) Si(gate)	1.84 × 10^4^–1.84 × 10^8^ particles/mL	2159 particles/mL		[116]
	CD63	Liquid-gate FETs	SiNW/APTES/GA/antibody(channel) Au(gate)	10^4^–10^10^ particles/mL	6.62 × 10^3^ particles/mL		[61]
	CD63	Liquid-gate FETs	Graphene/CDs/PBASE/ antibody(channel) Pt(gate)	10–1 × 10^7^ particles/μL	100 particles/μL		[117]
	CD63	Back-gate FETs	graphene/PBASE/ antibody(channel) Si(gate)	0.1–10 μg/mL	0.1 μg/mL		[118]
	CD63	Liquid-gate FETs	RGO/PASE/ antibody(channel) Ag/AgCl(gate)	3.3 × 10^4^–3.3 × 10^9^ particles/mL	33 particles/μL	Effectively distinguish between healthy individuals and prostate cancer patients	[23]
	CD9, CD81, CD63	Back-gate FETs	SiNW/APTES/GA/ antibody(channel) Si(gate)	3.80 × 10^4^–3.80 × 10^9^ particles/mL	1078 particles/mL		[74]
	CD9	Extended-gate FETs	Au/Cysteamine/Ti_3_C_2_T_x_ MXene/ aptamer(channel) MXene/Ti_3_C_2_T_x_(gate)	1 × 10^3^–1 × 10^7^ particles/mL	6.41 × 10^2^ particles/mL		[119]
	DBCO-functionalized exosome	Liquid-gate FETs	PEDOT:PSS/PLL-N_3_ (channel) Ag/AgCl(gate)	10^6^–10^9^ particles/mL	6 × 10^6^ particles/mL (LoI)		[67]
	CD91	Liquid-gate FETs	AgNPs/RGO/PBASE/antibody(channel) Ag/AgCl(gate)	1.2 × 10^3^–1.2 × 10^7^ particles/mL	1200 particles/mL	Effectively distinguish serum samples from healthy individuals and lung cancer patients	[27]
	PD-L1	Liquid-gate FETs	RGO/AuNPs/ aptamer(channel) Ag/AgCl(gate)	52.48 fg/mL–1 ng/mL	52.48 fg/mL	Effectively distinguish between healthy individuals and early-stage lung cancer patients; AUC = 1	[26]
	Hepatoma exosomes	Liquid-gate FETs	Graphene/PASE/artificial nucleotide aptamer(channel) Ag/AgCl(gate)	242 particles/mL–6.8 × 10^8^ particles/mL	242 particles/mL	Effectively distinguish serum samples from healthy individuals and patients with hepatocellular carcinoma	[120]
	EpCAM	Liquid-gate FETs	Graphene/PDA/ aptamer(channel) Au(gate)	112 particles/mL–3.63 × 10^8^ particles/mL	112 particles/mL	Effectively distinguish between healthy individuals and colon cancer patients	[121]
	EpCAM and TLS11a	Liquid-gate FETs	RGO/AuNPs/ aptamer(channel) Ag/AgCl(gate)	6 × 10^5^–6 × 10^9^ particles/mL	84 particles/μL	Effectively distinguish between healthy individuals and patients with hepatocellular carcinoma	[22]
	TMEM256, flotillin-2 and PSMA	Extended-gate FETs	ITO/SnO_2_/APTES/GA/antibody (channel) SnO_2_(gate)	100 fg/mL–1 pg/mL	100 fg/mL	Effectively distinguish between healthy individuals and patients with prostate cancer; AUC = 0.93	[30]
	NFL-specific of neuro- derived exosomes	Extended-gate FETs	USNWs /ITO/APTMS/ antibody(channel) SnO_2_(gate)	60 NDE/mL–6 × 10^14^ NDE/mL	60 NDE/mL	Effectively distinguish between healthy individuals and patients with Alzheimer’s disease	[122]
	Aβ and Tau proteins	Liquid-gate FETs	LiNbO_3_/F-IDTs (channel)	0.53 × 10^3^–10^9^ particles/mL	0.53 × 10^3^ particles/mL	Effectively distinguish between healthy individuals and AD patients	[123]
	CD63	Liquid-gate FETs	RGO/antibody(channel) Au/Cr(gate)	10^4^–10^6^ particles/mL			[124]
	GPC-1	Liquid-gate FETs	Graphene /TCPP/antibody(channel) Au(gate)	0.1 ng/μL–1000 ng/μL	0.1 ng/μL	Effectively distinguish between healthy individuals and patients with pancreatic ductal adenocarcinoma	[125]
	MUC1	Liquid-gate FETs	CNT/PLL/AuNPs/ Aptamer(channel) Ag/AgCl(gate)	1 fg/mL–100 pg/mL	0.34 fg/mL	Effectively distinguish between healthy individuals and patients with breast cancer; AUC = 0.94	[24]
exosome lysis	Aβ_42_	Liquid-gate FETs	RGO/AuNPs/TGA/antibody(channel) Ag(gate)	447 ag/mL–148 pg/mL	447 ag/mL	Effectively distinguish between healthy individuals and patients with Alzheimer’s disease; AUC = 1	[126]
	α-Syn	Liquid-gate FETs	CNT/PDA/ antibody(channel) Ag/AgCl(gate)	10 fg/mL–1 ng/mL	9.21 fg/mL	Effectively distinguish between healthy individuals and early-PD patients; AUC = 0.97	[127]
	miRNA10b	Liquid-gate FETs	RGO/PASE/PNA probe(channel) Ag/AgCl(gate)	1 × 10^−14^ M–1 × 10^−9^ M	78 fM	Effectively distinguish between healthy individuals and patients with pancreatic cancer; AUC = 0.98	[128]
	miRNA21	Liquid-gate FETs	CNT/AuNPs/ DNA probe (channel) Y_2_O_3_(gate)	1 aM–1 nM	0.87 aM	Effectively distinguish between healthy individuals and patients with breast cancer; AUC = 0.99	[129]
	miRNA21	Liquid-gate FETs	RGO/PLL/GQD/ PMO probe (channel) Ag(gate)	100 aM–1 nM	85 aM	Effectively distinguish between healthy individuals and patients with breast cancer	[130]
	miRNA-195 and miRNA-126	Extended-gate FETs	Au/DNA probe(channel) Au(gate)	1 fM–100 pM	84 aM for microRNA-195; 75 aM for microRNA-126	Effectively distinguish between healthy individuals and patients with breast cancer	[29]
	miRNA-106a	Back-gate FETs	IGZO/Al/AuNPs/DNA probe IGZO(channel) AlO_x_/Al/AlO_x_ (gate)	1 fM–1 μM	0.23 fM	Effectively distinguish between healthy individuals and patients with gastric cancer	[25]
	miRNA-196a	Liquid-gate FETs	Au/DNA probe(channel) Au (gate)	1 × 10^−18^ M–1 × 10^−8^ M	1.82 × 10^−19^ M	Effectively distinguish between healthy individuals and patients with pancreatic ductal adenocarcinoma; AUC = 0.98	[131]
	microRNAs from glioma exosome	Back-gate FETs	IGZO-TFT /APTES/DNA probe (channel) Ti (gate)	1 fM–100 pM	350 aM	Effectively distinguish between healthy individuals and patients with glioma	[31]
	Aβ_1–40_, Aβ_1–42_, t-tau, and p-tau^181^ proteins	Liquid-gate FETs	Au microelectrode array (MEA)/3-MPA + 11-MUA (channel) Au(gate)	Aβ_1–42_: 10^−18^–10^−12^ M Aβ_1–40_: 10^−18^–10^−11^ M t-tau: 10^−19^–10^−12^ M p-tau^181^: 10^−19^–10^−13^ M	Aβ_1–42_: 1 aM Aβ_1–40_: 1 aM t-tau: 1 zM p-tau^181^: 1 zM	Effectively distinguish between healthy individuals and AD patients; AUC = 1(Combined detection of the four AD core biomarkers)	[132]

**Table 2:** Comparison of intact-exosome and lysate-based FET detection strategies.

Dimension	Intact-Exosome Detection	Lysate-Based Detection
Target	Whole vesicles (30–150 nm) bearing membrane markers	Released miRNA or protein cargo
Mechanism	Electrostatic + EDL restructuring	Direct electrostatic gating
LOD	~60–10^5^ particles/mL	aM–fM (nucleic acids); ag–fg/mL (proteins)
Information content	Particle counts and marker co-expression on the same vesicle	Molecular concentration of specific cargo
Best-fit applications	Screening, subtype classification, tumor-derived exosome identification	Precision diagnosis, early-stage detection, treatment monitoring

## Data Availability

Data sharing is not applicable to this article as no new data were created or analyzed in this study.
